# Multifaceted Roles of Neutrophils in Autoimmune Diseases

**DOI:** 10.1155/2019/7896738

**Published:** 2019-03-20

**Authors:** Yi Zhao, Tony N. Marion, Qian Wang

**Affiliations:** ^1^Department of Rheumatology & Immunology, West China Hospital, Sichuan University, Chengdu, China; ^2^Department of Microbiology, Immunology and Biochemistry, University of Tennessee Health Science Center, Memphis, TN, USA; ^3^Department of Rheumatology, Peking Union Medical College Hospital, Peking Union Medical College & Chinese Academy of Medical Science, Beijing, China

Neutrophils, also known as polymorphonuclear leukocytes, are the most abundant type of granulocytes. As the most abundant type of white blood cells in most mammals, neutrophils are an essential barrier for host defense. Impaired development, maturation and function and death of neutrophils lead to abnormal conditions in the immune system, which can initiate autoimmune disorders. In response to infection or injury, neutrophils are recruited by chemokines and cytokines to areas of pathogen exposure and/or tissue damage where pathogen-associated molecular patterns (PAMPs) and/or damage-associated molecular patterns (DAMPs), respectively, engage and stimulate the infiltrating neutrophils. Neutrophils acquire distinct and different phenotypic functions in response to stimulation that may include phagocytosis, degranulation, ROS production, and release of neutrophil extracellular traps (NETs) to impede pathogens or clear sterile inflammatory stimuli.

In recent years, neutrophils have emerged as players in the pathogenesis of various systemic autoimmune diseases and autoinflammatory syndromes, such as adult-onset Still's disease (AOSD) driven by innate immunity and rheumatoid arthritis (RA), systemic lupus erythematosus (SLE), and ANCA-associated vasculitis (AAV) driven by adaptive immunity. The precise role of neutrophils in inflammatory autoimmune disease is generally poorly understood. Research advances in neutrophil biology and elucidation of the role of neutrophils in systemic autoimmune disease will hopefully not only better define how neutrophils contribute to autoimmune disease but also identify potential novel therapeutic targets for treatment of inflammation and autoimmune disease.

The multifaceted role of neutrophils refers not only to how neutrophils contribute to pathogenesis in autoimmune disorders but also to how their functions are essential for the elimination of pathogens and sterile stimuli, such as nanoparticles, in health or disease conditions. In this regard, P. Yang et al. have reviewed the heterogeneous phenotypes and functions of neutrophils and discussed their flexibility and elasticity and their presentation of “different faces” in response to various disease states including autoimmunity and autoinflammation as well as cancer. H. Yang et al. have reviewed how neutrophils engage and respond to different types of nanoparticles. This topic is timely and germane to recent research and development of nanoparticles as drug delivery vehicles, each with varying potential to initiate sterile inflammation. Depending upon structure and size, nanoparticles can easily penetrate extracellular matrix barriers and engage the innate immune system. This review also summarizes NETs formation induced by various types of nanoparticles, for example, gold, silver, polystyrene, and nanodiamond. The authors also provide insight about how NETosis triggered by different nanoparticles may facilitate initiation and resolution of inflammatory responses.

RA is a chronic inflammatory autoimmune disease characterized by autoantibodies and systemic inflammation manifested as chronic synovitis, bone erosion, and articular deformity. The presence of autoantibodies against citrullinated protein antigens is thought to be a major cause of disease. It has been speculated that citrullinated proteins in RA were generated from neutrophils during NETosis. C. L. Holmes et al. determined that depending upon the stimulus, human or mouse neutrophils produced citrullinated, uncitrullinated, or mixed citrullinated and uncitrullinated NETs *in vitro*. Interestingly, monosodium urate (MSU) crystals and *Candida albicans* induced mostly citrullinated NETs in human-isolated neutrophils. In addition, PAD4 but not PAD2 was indispensable for citrullinated proteins in NETs. W. Chen et al. reviewed neutrophil functions in RA by discussing updated concepts and regulators of neutrophil migration in RA inflammatory conditions. As a supposed initiating factor of RA, potential role of NETosis in autoantibody production was also reviewed as decision-making for therapeutic strategies for RA.

Evidence indicates that neutrophil infiltration is involved in the pathophysiology of various autoimmune diseases. Inhibition of neutrophil migration may be essential as a target for treatment of autoimmune disorders. M. V. Jones and M. Levy identified a CXCR2 antagonist as a potential inhibitor of neutrophil migration in neuromyelitis optica animal models. The potential for CXCR2 inhibitors to reduce inflammation in experimental animal models, including for neuromyelitis optica, merits further investigation. K. Orczyk and E. Smolewska tested S100A12 levels in patients with juvenile idiopathic arthritis and defined S100A12 as a potential diagnostic biomarker and prognostic indicator for juvenile idiopathic arthritis.

A consequence of immune evolution, especially for innate immune function, has been a selective emphasis for speed in recognizing and responding to potential pathogens. The replicative vigor of pathogens presents the evolutionary rationale for this selection. The emergence of PAMPs and DAMPs as the recognition signals for pathogen presence is further evidence for emphasis on speed to detect pathogens. PAMP and DAMP recognition is inherent. Neither PAMPs nor DAMPs need to be modified to be detected by innate immune cells or proteins. This system for pathogen recognition is inherently dangerous since the same system for pathogen recognition can mistakenly identify necessary self-molecules and tissues as having potential pathogenic origin. The outcome of that “mistake,” of course, can be damaging inflammation and/or autoimmunity. Neutrophils are critical early in the pathogen recognition process and, consequently, critical to immune protection and potential immune damage. The reports included in this special issue document the latter and illustrate the critical role of neutrophils in autoimmune disease.

